# Porous‐Structure‐Promoted Tribo‐Induced High‐Performance Self‐Powered Tactile Sensor toward Remote Human‐Machine Interaction

**DOI:** 10.1002/advs.202203510

**Published:** 2022-09-08

**Authors:** Li Su, Quan Xiong, Haoyu Wang, Yunlong Zi

**Affiliations:** ^1^ Hebei Key Laboratory of Micro‐Nano Precision Optical Sensing and Measurement Technology School of Control Engineering Northeastern University at Qinhuangdao Qinhuangdao Hebei 066004 China; ^2^ Department of Biomedical Engineering National University of Singapore Singapore 117583 Singapore; ^3^ Department of Mechanical and Automation Engineering The Chinese University of Hong Kong Shatin, New Territories Hong Kong China; ^4^ Thrust of Sustainable Energy and Environment The Hong Kong University of Science and Technology (Guangzhou) Nansha Guangdong 511400 China; ^5^ HKUST Shenzhen‐Hong Kong Collaborative Innovation Research Institute Futian Shenzhen Guangdong 518048 China; ^6^ Department of Mechanical and Aerospace Engineering The Hong Kong University of Science and Technology Clear Water Bay Hong Kong SAR China

**Keywords:** porous‐structure, remote human‐machine interaction, tactile sensors, tribo‐induced devices

## Abstract

Self‐powered tactile sensor with versatile functions plays a significant role in the development of an intelligent human‐machine interaction (HMI) system. Herein, a hybrid self‐powered porous‐structured tactile sensor (SPTS) is proposed by monolithically integrating a porous triboelectrification‐induced electroluminescence (TIEL) component and a single‐electrode triboelectric nanogenerator with the high charge generation in the bulk volume. At a low pressure of 10 kPa, TIEL intensity can be significantly improved by three times, which is superior to that in previous reports, with enhanced triboelectricity. Based on the enhancement brought by the porous structure and optimized parameters, the SPTS achieves significant sensing performance in both optical and electrical modes. To demonstrate the potential of practical applications, a programmable optical and electrical dual‐mode HMI system is established based on SPTS to remotely control an intelligent vehicle and operate a computer game through identifying finger touch trajectories. This work not only contributes a new economical‐effective methodology toward a high‐performance tribo‐induced self‐powered tactile sensor but also facilitates the remote control of HMI with dual‐mode functionality, which has broad potential applications in the fields of intelligent robots, augmented reality, flexible wearable electronics, and smart home.

## Graphical abstract

An all‐in‐one self‐powered porous‐structured tactile sensor (SPTS) is developed with ultrasensitive sensing performances. The SPTS is successfully demonstrated in remote control of the human‐machine interaction system by both electrical and optical signals, which has broad potential applications in the sensor network in Internet of Things.

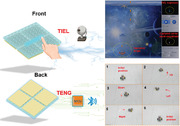

## 1 Introduction

Accompanied by the rapid development of artificial intelligence and 5G technology, human‐machine interaction (HMI) bridging the human and machines plays an essential role in the popularization of the Internet of Things (IoT).^[^ ^1^, ^2^, ^3^, ^4^, ^5^ ^]^ In general, tactile sensing represents a key step in the transformation of tactile actions into electrical signals for machines.^[^ ^6^, ^7^, ^8^, ^9^, ^10^ ^]^ Despite plenty of research focusing on the spatial mapping of tactile sensing through electric readout, a major barrier toward practical application exists due to drawbacks such as high energy consumption, structural complexity, and high cost.^[^ ^11^, ^12^, ^13^, ^14^, ^15^ ^]^ Therefore, various optical intelligent materials with high spatial resolution and intuitive manner have attracted extensive attention to long‐distance wireless transmission in HMI application.^[^ ^16^, ^17^, ^18^, ^19^, ^20^, ^21^ ^]^ Among them, triboelectrification‐induced electroluminescence (TIEL) enabled by the coupling effect of triboelectrification and electroluminescence (EL) has demonstrated various desirable merits, such as non‐destruction, high responsivity, and low stress threshold, compared with the conventional mechanoluminescence (ML).^[^ ^22^, ^23^, ^24^, ^25^ ^]^ Therefore, TIEL has demonstrated potentials in a wide range of applications including anti‐counterfeiting,^[^ ^26^, ^27^, ^28^ ^]^ real‐time vision sensor,^[^ ^29^, ^30^, ^31^ ^]^ HMI display,^[^ ^32^, ^33^ ^]^ and self‐powered illumination.^[^ ^34^, ^35^, ^36^ ^]^ However, the intensity in TIEL is still not high enough, which turns into a major challenge hindering its practical applications. The existing technologies intended to improve TIEL intensity are still limited.

In addition, novel technologies that enable transmission and reception of optical‐electrical dual readout information under tactile stimuli are in the spotlight recently. The described dual‐mode tactile sensing system has mainly concentrated on ML^[^ ^22^, ^37^ ^]^ and TIEL.^[^ ^17^, ^38^ ^]^ Although inspiring, it is extremely tough due to the difficulties in integrating the different functional components and limited performance. To date, the porous structure has been commonly applied to improve the energy harvesting efficiency of the triboelectric nanogenerator (TENG) with the advantages of facile and prompt fabrication.^[^ ^39^, ^40^, ^41^ ^]^ The contact of porous materials with cavities can achieve contact electrification at the microscale, thus improving the triboelectric charge transfer substantially.^[^ ^42^, ^43^ ^]^ Since both TIEL and TENG can be triggered by triboelectrification, the high‐performance tribo‐induced device is expected to be achieved by a built‐in porous structure in optical‐electrical dual modes by simultaneously utilizing a large number of tribo‐charges generated in the bulk volume. In addition, by taking the TIEL signal as an efficient optical signal channel for accurate remote control, the application scope of intelligent optical materials in the sensor network in IoT can be further broadened.

In this work, a self‐powered porous‐structured tactile sensor (SPTS) is developed by a porous TIEL component with a back electrode. The TIEL component is fabricated by ZnS:Cu,Al EL phosphors distributed in a porous polydimethylsiloxane (PDMS) matrix. Since the electric field variation of EL phosphors can be significantly promoted by the deformation of inner cavities, TIEL intensity is enhanced by three times as compared to the dense tactile sensor (DTS) without cavities, which is superior compared to previous reports. In the meantime, the electrostatically induced charge in the back electrode can be also enhanced, which is available as an alternative signal channel. Based on the enhancements brought by the porous structure and optimized parameters, the SPTS (3.0 × 3.0 × 0.1 cm^3^) demonstrates excellent sensing performance, including ultralow detection limit (1 kPa), ultrahigh sensitivity (0.2 kPa^−1^ and 22 V kPa^−1^), ultrafast response time (<8 ms) as well as superior durability and stability (>20 000 cycles). To demonstrate its practical application in tactile sensing through identifying finger touch trajectories, an intelligent vehicle is remotely controlled by the output signal from the back electrode. Moreover, the video game is operated by TIEL optical signals processed by a self‐developed software. Therefore, this novel SPTS demonstrates the dual‐mode capability of triggering remote HMI applications, which may provide unique opportunities for its applications in the fields of intelligent robots, augmented reality, flexible wearable electronics, smart homes, etc.

## 2 Results and Discussion

**Figure 1:**
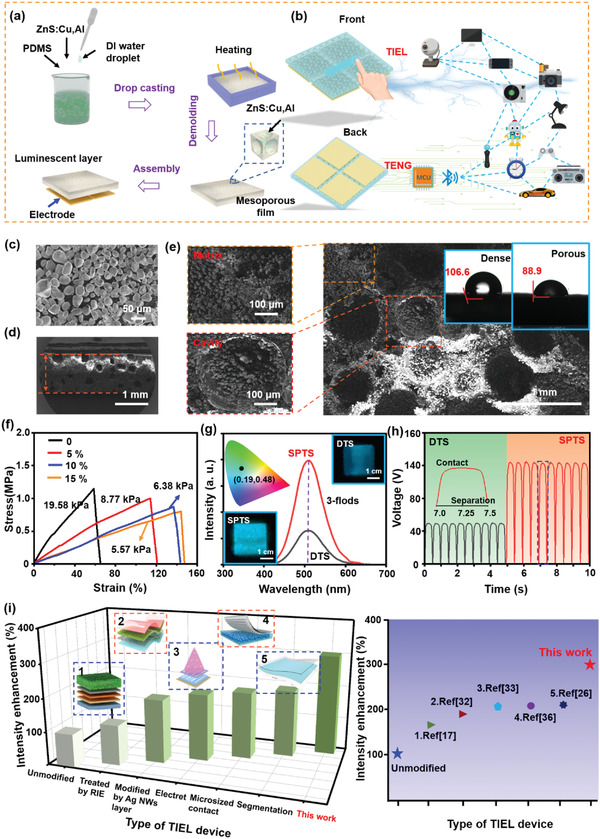
Schematic diagram of the fabrication process and characterization of SPTS. a) Schematic diagram of device fabrication. b) Conceptual diagram of SPTS, which is capable of converting the touch stimulation into visible light and electrical signals through TIEL and TENG processes for the control of various consumer electronics. c) SEM image of ZnS:Cu,Al particles. d) Cross‐sectional SEM image of the porous luminescent layer. e) Surface SEM image of the porous luminescent layer. Left section: the enlarged view of the matrix (upper) and the cavity (bottom). Inset: different water contact angles from porous and dense luminescent layers, respectively. f) Stress–strain curve of SPTS affected by the porosity. g) TIEL intensity of SPTS and DTS (3.0 × 3.0 × 0.1 cm^3^) at 10 kPa under sliding mode. Inset: CIE chromaticity coordinate diagram of the emission spectrum and the corresponding luminescent photographs. h) Voltage output of SPTS and DTS at 10 kPa under sliding mode. Inset: the corresponding voltage output with one complete cycle. i) Comparison of the enhanced TIEL intensity in this work and previous studies.

The schematic diagram for the fabrication procedure of SPTS based on the emulsion template method is illustrated in **Figure** **1**a and detailed in the Experiment Section. Featuring a double‐layer structure, the SPTS is integrated by a porous luminescent layer (Figure S1, Supporting Information) and a back electrode layer. The application scenario of SPTS is shown in Figure 1b. Upon tactile stimulation, the optical TIEL signal can be generated by triboelectrification and EL without any external power supply. At the same time, the electrical output signal from the back electrode can be generated through the electrostatic induction of the triboelectric charge, serving as an additional signal channel for remote control. With the assistance of programmed microcontroller unit (MCU) or self‐developed software, these signals can be regarded as control commands for various consumer electronics. Figure 1c presents the scanning electron microscopy (SEM) images of ZnS:Cu,Al phosphors with an average diameter of ≈40 µm. The X‐ray diffraction (XRD) pattern and energy‐dispersive spectroscopy (EDS) are shown in Figure S2, Supporting Information to confirm the composition of the material. In this case, ZnS:Cu,Al phosphors are chosen due to its higher luminous brightness than commercial ZnS: Cu because it is co‐doped with Al as a co‐activator, as shown in Figures S3 and S4, Supporting Information. The cross‐sectional SEM image of the porous luminescent layer is displayed in Figure 1d, revealing a sponge‐like structure with a thickness of ≈1 mm. The SEM image shows the surface of the porous luminescent layer in Figure 1e, indicating that adjacent cavities (with an average diameter of 40 µm) are segmented by the walls made up of ZnS‐PDMS composites. The left insets show the zoomed‐in images of a matrix and a cavity, respectively, reflecting that ZnS:Cu,Al particles are distributed both inside the matrix and on the surface. This plays an essential role in promoting triboelectric charge generation, and will be discussed later. The upright insets show that the water contact angle of the film is reduced by pores on the surface. Hence, as suggested by the results of SEM images, there are numerous cavities both on the surface and inside the SPTS. Moreover, the stress–strain curves of porous luminescent layers (2.0 × 1.0 × 0.1 cm^3^) are shown in Figure 1f, which indicates that Young's modulus is affected by the porosity defined as the volume ratio of pores in the whole layer (calculated in Note 1, Supporting Information). According to the experimental results, the increase of porosity is accompanied by the progressive elongation of the film and the reduction of the elastic modulus. To quantify luminescence intensity and electric output, a test platform was constructed, as shown in Figure S5, Supporting Information. Figure 1g displays that the TIEL intensity of the SPTS (3.0 × 3.0 × 0.1 cm^3^) was three times higher than that of the DTS with the same peak position when thermoplastic polyurethane (TPU) was taken as an external object at the pressure of 10 kPa under the sliding stimulus (Figure S6 and Movie S1, Supporting Information). The inset presents that the CIE coordinate of TIEL is (0.19, 0.48), corresponding to blue‐green light. Meanwhile, the output voltage of SPTS reaches up to 120 V, which is found to be significantly superior to DTS (50 V), as shown in Figure 1h. These results suggest that the SPTS can generate higher TIEL intensity and electrical output than the DTS without cavities. Compared with previously reported TIEL, such as that with the enhancement brought by RIE etching,^[^ ^17^ ^]^ Ag‐NWs layer modification,^[^ ^32^ ^]^ the addition of electret,^[^ ^33^ ^]^ micro‐contact,^[^ ^36^ ^]^ and segmentation,^[^ ^26^ ^]^ the performance of SPTS is superior, with three times higher TIEL intensity than that of the dense material‐based device, as shown in Figure 1i. Additionally, the SPTS based on different EL phosphors can display a variety of luminescent colors (green‐blue, green, and orange), whereas the ZnS:Cu,Al‐based SPTS shows the highest TIEL intensity and responsivity among them, indicating that the SPTS can sense external stimuli in real‐time via visually bright and colorful luminescence (Figure S7, Supporting Information).

**Figure 2:**
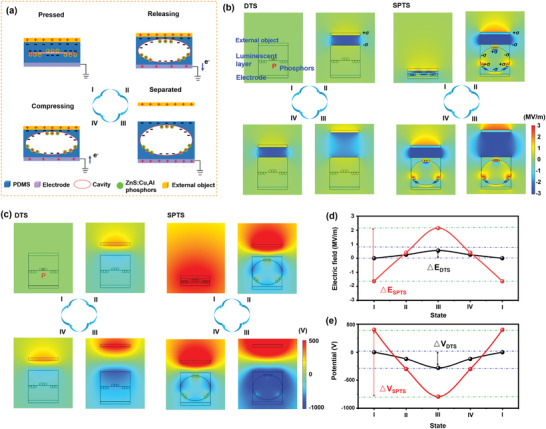
The working mechanism of SPTS. a) Simplified diagrams showing the process of TIEL and electricity generation process of SPTS under contact‐separation mode. The 2D models of b) electric field and c) potential distribution with the cross‐section of DTS and SPTS in the four typical working states as constructed using COMSOL software. d) The extracted electric field and e) potential value of point “P” on the phosphor in the four typical working states of DTS and SPTS in the simulation.

The working mechanisms of the SPTS under the typical contact‐separation and sliding working modes are schematically illustrated in **Figure** **2**a and Figure S8, Supporting Information, respectively, which were achieved based on the working principle of triboelectrification, EL effect, and electrostatic induction. Due to the similarity of the charge transfer behaviors of contact‐separation and sliding modes in the basic principle of triboelectrification, the contact‐separation working mode was used to investigate the working mechanism systematically.^[^ ^17^ ^]^ In state I, the cavity is compressed when the pressure is loaded onto the SPTS, leading to the contact electrification between ZnS:Cu,Al phosphors, and the PDMS matrix on the inner cavity wall. In this regard, positive and negative charges are generated on their surfaces, respectively. In state II, the pressure is released and phosphors are separated from the PDMS matrix, resulting in charge separation. Then, a current is generated between the back electrode and the ground due to electrostatic induction, until the external object is completely separated (state III). A reverse current is subsequently produced in the external circuit as the object approaches the surface and the pressure is loaded once again. Consequently, phosphors come into contact with the PDMS matrix again (state IV). In this circumstance, the varying electric field generated inside the device can excite the EL of phosphors in the PDMS matrix, along with the electric current. To verify the above‐mentioned working mechanism, COMSOL software was applied to simulate the electric field and potential distribution of DTS and SPTS in one complete working cycle, as shown in Figure 2b,c, and Note 2, Supporting Information, which kept identical absolute charge density. It is thus shown that the electric field and potential variation (ΔE and ΔV) of phosphors in the DTS (at point P) are only 0.55 MV m^−1^ and 280 V in a period, respectively (Figure 2d), compared to 3.8 MV m^−1^ and 1200 V in the SPTS (Figure 2e). The results demonstrate the significant improvement of the electric field through the porous structure. The significantly increased electric field variation in the inner cavities causes a stronger intensity of EL than that in the matrix (Figure 1h and Movie S1, Supporting Information). Furthermore, the electrode potential in SPTS is also evidently higher than that in DTS (Figure S9, Supporting Information), which is consistent with the electric output measured from DTS and SPTS, as shown in Figure 1h. Therefore, the simulation results provide compelling evidence for the working mechanism as illustrated in Figure 2a. As a result, SPTS demonstrates higher TIEL intensity and greater electrical output than that of DTS through the porous structure.

**Figure 3:**
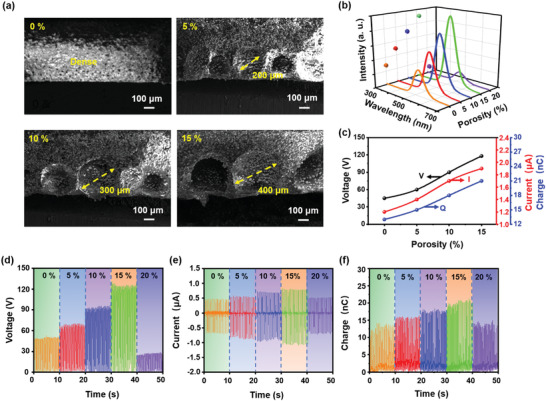
The impact of porosity on the TIEL intensity and electrical output of SPTS. a) Cross‐sectional SEM images of SPTSs (3.0 × 3.0 × 0.1 cm^3^) at different levels of porosity with the same ZnS content of 40 wt%. b) TIEL intensity and c) electric output of SPTS (3.0 × 3.0 × 0.1 cm^3^) under a cycled sliding pressure of 10 kPa. The corresponding d) voltage, e) current, and f) transferred charge, respectively.

To identify critical parameters that affect the performance, an in‐depth investigation was conducted about the impact of porosity, ZnS content, and porous luminescent layer thickness on the TIEL intensity and electrical output. First, the impact of porosity was studied. In case of maintaining the identical volume of 3.0 × 3.0 × 0.1 cm^3^ and the same ZnS content of 40%, multiple SPTSs were fabricated with the porosity gradually increasing from 0 to 15%, corresponding to the cavity diameter of 0 to 400 µm, as revealed by the SEM images in **Figure** **3**a, which is in high agreement with previous reports.^[^ ^44^ ^]^ The measured TIEL intensity of fabricated SPTSs is presented in Figure 3b. Moreover, the electrical output of the SPTSs exhibits a similar trend to TIEL intensity, as shown in Figure 3c. Both the TIEL intensity and the electrical output witness an increase at a low porosity of 0 to 15%, and then an abrupt decrease at a high porosity of 20%, which can be attributed to the deterioration in the film quality caused by the saturated mixture of ZnS‐PDMS/DI water suspension.^[^ ^45^ ^]^ The corresponding output voltage, current, and transferred charge are presented in Figure 3d,e,f, respectively. With the optimized porosity, the output can be greatly improved from 45 V, 1.2 µA, and 13 nC at 0% to 117 V, 1.9 µA, and 21 nC at 15%. These results suggest that the increase of porosity within a certain range facilitates the charge generation inside the deformed cavities, which double confirms our proposed mechanism.

**Figure 4:**
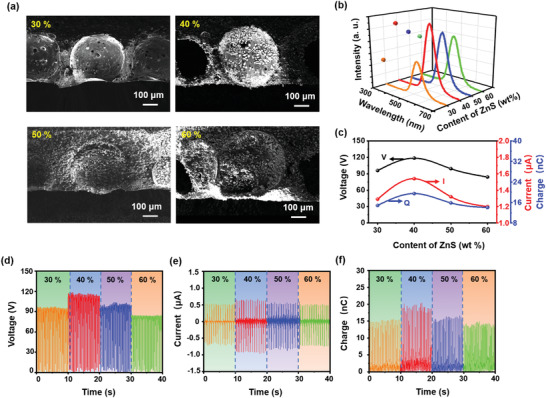
The impact of the content of ZnS:Cu,Al particles on the TIEL intensity and electrical output of SPTS. a) Cross‐sectional SEM images of SPTSs (3.0 × 3.0 × 0.1 cm^3^) at different content of ZnS:Cu,Al with a constant porosity of 15%. b) TIEL intensity and c) electric output under a cycled sliding pressure of 10 kPa. The corresponding d) voltage, e) current, and f) transferred charge, respectively.

Second, the content of ZnS:Cu,Al particles also substantially affects the TIEL intensity and electrical output. **Figure** **4**a shows the cross‐sectional SEM images of multiple porous luminescent layers (3.0 × 3.0 × 0.1 cm^3^) with varying amounts of ZnS:Cu,Al particles at a constant porosity of 15%. When the content of ZnS particles reaches 40 wt%, TIEL intensity and electrical output both reach the maximum value as shown in Figure 4b,c, respectively. The corresponding output voltage, current, and transferred charge are presented in Figure 4d,e,f, respectively. The optimal content of SPTS ensures an appropriate number of particles on the inner cavity's walls for contact electrification between ZnS and PDMS. In contrast, the excessively high content of particles tends to reduce the area of PDMS and suppress tribo‐charge generation. At the same time, the mechanical properties of the matrix will deteriorate because of the difficulty in transferring stress effectively caused by poor interfacial compatibility.^[^ ^41^ ^]^ As a result, the TIEL intensity and electrical output are both decreased.

**Figure 5:**
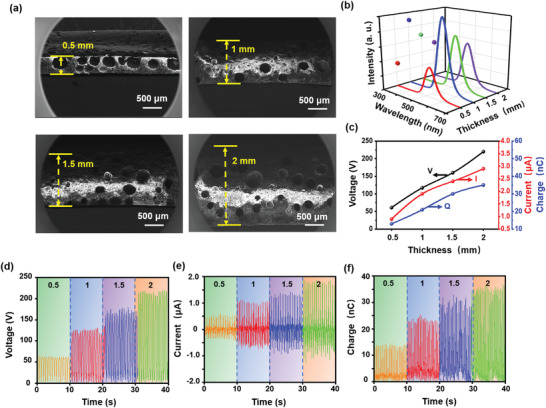
The impact of the porous luminescent layer thickness on the TIEL intensity and electrical output of SPTS. a) Cross‐sectional SEM images of the porous luminescent layer of SPTSs (3.0 × 3.0 cm^2^) with different thicknesses (all samples have the constant porosity of 15% and the same content of ZnS particles of 40 wt%). b) TIEL intensity and c) electric output under a cycled sliding pressure of 10 kPa. The corresponding d) voltage, e) current, and f) transferred charge, respectively.

TIEL intensity and electric output are affected by the thickness of the porous luminescent layer as well. Multiple SPTSs (3.0 × 3.0 cm^2^) with the luminescent layer thickness increased by the increment of 0.5 mm were fabricated (**Figure** **5**a) at the identical porosity of 15% and ZnS particles’ content of 40 wt%. The TIEL intensity of SPTS reaches the maximum when the thickness reaches 1 mm (Figure 5b). Figure 5c presents the electrical output of SPTSs increasing with thickness, which behaves differently from the TIEL intensity. The corresponding output voltage, current, and transferred charge are illustrated in Figure 5d,e,f, respectively, revealing that the electrical output increases progressively from 61 V, 0.9 µA, and 13 nC to 220 V, 2.9 µA, and 35 nC. The increased thickness allows more cavities as sites for triboelectrification, thereby reinforcing the tribo‐charge generation. However, the excessively thick layer may block the escape of the emitted light, leading to the weakened TIEL intensity without affecting the electrical output. On this basis, the optimal thickness is set to 1 mm with the maximum TIEL intensity and reasonable electrical output.

**Figure 6:**
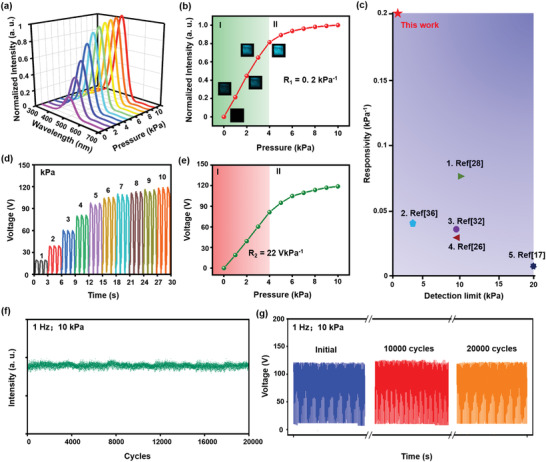
The sensing performance of the SPTS. a) TIEL intensity and d) voltage output of the SPTS (3.0 × 3.0 × 0.1 cm^3^) at different sliding pressures ranging from 1 to 10 kPa. b) TIEL intensity and e) voltage output pressure–response curves in (a) and (d), respectively. Inset of (b): The corresponding luminescent photographs of TIEL. c) Comparison between the responsivities and detection limits of TIEL intensity in this work and previous studies. The cyclic measurement of f) TIEL intensity and g) voltage output of the SPTS at the frequency of 1 Hz and the pressure of 10 kPa.

Based on the above‐optimized parameters, the sensing performance of the SPTS was investigated carefully under the sliding mode. The measured TIEL intensity and output voltage of SPTS (3.0 × 3.0 × 0.1 cm^3^) in response to the varied loading pressure from 1 to 10 kPa are shown in **Figure** **6**a,d at 1 Hz. Meanwhile, the corresponding current and transferred charges are presented in Figure S10, Supporting Information. Because of the enhanced contact area during the deformation of cavities under increased pressure, it is noticeable that TIEL intensity and output voltage both largely depended on the pressure at the contacted surface. Besides, their plotted pressure–response curves almost present a similar changing trend (Figure 6b,e). This observation further validated the proposed triboelectrification mechanism and ruled out other possible mechanisms. The pressure responsivity of TIEL intensity (*R_1_*) was defined as ΔI/ΔP, where ΔI is the variation of TIEL intensity and ΔP represents pressure increment, showing different responsivities in two distinct stages (region I from 0 to 4 kPa and II from 4 to 10 kPa). *R_1_* can be calculated as 0.2 kPa^−1^ in the pressure region below 4 kPa (Figure 6b), which is one of the highest reported responsivities in available literature (Figure 6c). The pressure responsivity of output voltage (*R_2_*) characterized by ΔV/ΔP in the pressure region of below 4 kPa also exhibited a high value of 22 V kPa^−1^, as shown in Figure 6e. Thus, the SPTS can endow versatile functions which are greatly sensitive in both optical and electrical modes. More importantly, even an extremely low pressure of 1 kPa was sufficient to induce substantial luminescence so that the SPTS possessed one of the lowest detection limits compared with those in other recent available reports (Figure 6c and Movie S2, Supporting Information). It was found that TIEL intensity almost linearly relates to the velocity until 5 cm s^−1^ (Figure S11, Supporting Information) and shows a fast response time of less than 8 ms (Figure S12, Supporting Information). Moreover, the cyclic measurements of TIEL intensity and output voltage remain remarkable during and after 20 000 cycles at the pressure of 10 kPa and the frequency of 1 Hz, suggesting the excellent long‐term stability and durability of the SPTS, as illustrated in Figure 6f,g. Such excellent sensing performance ensures that the SPTS is of great significance to acquire reliable real‐time sensing response in practical HMI applications.

**Figure 7:**
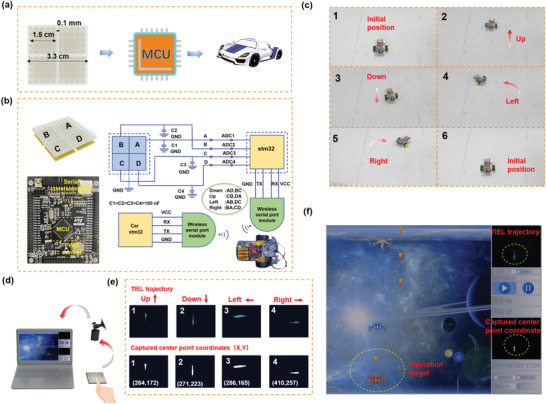
HMI application of SPTS. a) Schematic diagram of remote control on an intelligent vehicle using the electrical signal received from TENG with the assistance of a programmable MCU. b) Complete flowchart of the signal processing. c) The corresponding photograph showing the movement of the intelligent vehicle. d) Schematic diagram of operational computer games through TIEL characteristics with the assistance of a camera and a self‐developed software. e) The corresponding TIEL trajectory images and the captured center point coordinates. f) The photograph of the computer game interface.

Benefitting from unique sensing performance, the SPTS‐based programmable HMI system was established to identify finger touch trajectories (Figure S13, Supporting Information) for achieving the remote control of commercial electronics and demonstrate practical applications in tactile sensing. First, an intelligent vehicle was remotely controlled by combining the electrical signal received from the back electrode of the SPTS with a MCU (STM32F407, programmed by LabVIEW), as illustrated in **Figure** **7**a. Details and flowcharts of the signal processing are shown in Figure 7b. By splitting the material into four separate channels (A, B, C, D) with back electrodes, the MCU controller can determine the sliding direction of the finger according to the time sequence of the detected voltage signal for each channel. Then, the direction information is forwarded to the vehicle (through another MCU) by a wireless serial port, and the rotational speed of two rear‐wheel motors is controlled by adjusting the duty cycle of plus width modulation, to operate the motion direction of the vehicle, as shown in Figure 7c and Movie S3, Supporting Information. In addition, with the assistance of a camera and a self‐developed software (programmed by LabVIEW), a protagonist plane in the video game was controlled to elude attacks and shoot enemy planes, as illustrated in Figure 7d. Depending on the movement trajectory captured by the camera, the plane shifts accordingly in the game, as shown in Movie S4, Supporting Information. To sum up, the SPTS demonstrates the capability to trigger the remote HMI via both electrical and optical signals.

## 3 Conclusion

In summary, an all‐in‐one SPTS was developed by integrating a porous luminescent layer with a back electrode based on the coupling effect of triboelectrification, EL effect, and electrostatic induction. The TIEL intensity of the SPTS (3.0 × 3.0 × 0.1 cm^3^) was three times higher than that of the DTS under the same mechanical pressure of 10 kPa, which was superior to that in previous reports, and output voltage increased from 45 V and 1.2 µA to 117 V and 1.9 µA, respectively. The results of systematic experiments and theoretical simulations confirm that the improvement of TIEL intensity and electrical output is ascribed to the increased electric field variation caused by the triboelectrification in the inner deformed cavities. The SPTS demonstrates ultrasensitive sensing performances including ultralow detection limit, ultrahigh sensitivity, ultrafast response, and excellent stability and durability. In addition, a relationship among TIEL features, electric output, sensing performance, and deformation was also investigated. Moreover, the SPTS has been successfully demonstrated in remote control of the HMI system by both electrical and optical signals. Establishing a new methodology of remotely controlling consumer electronics through dual‐mode functionality, this novel SPTS has broad potential HMI applications in the fields of robots, augmented reality, smart homes, and flexible wearable electronic devices.

## 4 Experimental Section

### Fabrication Process of an SPTS

PDMS (Sylgard 184, Dow Corning Co.) was purchased for fabricating porous elastomeric composites. ZnS:Cu,Al powders (Ke Yan Co.) were purchased as the EL phosphors. The ZnS:Cu,Al phosphors were first mixed with the PDMS matrix until they were well blended. Subsequently, the PDMS curing agent (with a curing‐agent‐to‐base ratio of 1:10) was added to the mixture, which was then uniformly dispersed immediately. Aftward, DI water was added as the emulsion template. To create different porosity, the mass ratio between DI water to ZnS‐PDMS composite was adjusted. Then, the precursor mixture was stirred again by centrifugation at 2000 r for 10 min, thereby obtaining a uniformly‐mixed water suspension. After being poured onto the template with a specific size, the prepared material was placed in the vacuum drying oven at 80 °C for 24 h to completely remove the DI water template and obtain a solidified porous luminescent film. At last, the full SPTS was obtained after assembly with a copper electrode.

### Measurements

All SEM and EDS images were captured by field emission scanning electron microscope (Nova Nano 450, FEI). The crystal structure of ZnS was characterized by X‐ray diffraction at room temperature (D8 Advance, Bruker AXS). The optical emission was observed using a spectrometer and a vertically arranged optical fiber collimating lens spectrometer (Nova, Idea optics). The electrical output of the material was measured by an electrometer (Keithley 6514, Tektronix). The assembly of a linear motor (E1250, Lin Mot‐E) and a pressure sensor (M5, Mark‐10) were applied to test the optical emission and electrical output. The stress‐strain test was conducted in a microcomputer‐controlled electronic universal material testing machine (CTM2050, CTM).

All volunteers have known all details about the experiment which was touching the sensor with finger to detect the electric and optical signals. The experiment results will be used to conduct further research. Hebei Key Laboratory of Micro‐Nano Precision Optical Sensing and Measurement Technology will ensure the health and safety of all volunteers in the experiment. All volunteers have agreed to participate in the experiment.

## Conflict of Interest

The authors declare no conflict of interest.

## Supporting information

Supporting Information

Supplemental Movie 1

Supplemental Movie 2

Supplemental Movie 3

Supplemental Movie 4

## Data Availability

The data that support the findings of this study are available from the corresponding author upon reasonable request.
