# Supplementary material for: Porous‐Structure‐Promoted Tribo‐Induced High‐Performance Self‐Powered Tactile Sensor toward Remote Human‐Machine Interaction
Source: Adv Sci (Weinh). 2022 Sep 8;9(32):2203510. doi: 10.1002/advs.202203510 (PMC9661844; doi:10.1002/advs.202203510)
Supplement: Supplementary file 1 — Supporting Information [file ADVS-9-2203510-s004.pdf]

ADVANCED SCIENCE
Open Access

Supporting Information

for *Adv. Sci.*, DOI 10.1002/advs.202203510

Porous-Structure-Promoted Tribo-Induced High-Performance Self-Powered Tactile Sensor toward Remote Human-Machine Interaction

*Li Su, Quan Xiong, Haoyu Wang and Yunlong Zi**

Supporting Information

Porous-Structure-Promoted Tribo-Induced High-Performance Self-Powered Tactile Sensor toward Remote Human-Machine Interaction

*Li Su, Quan Xiong, Haoyu Wang, and Yunlong Zi**

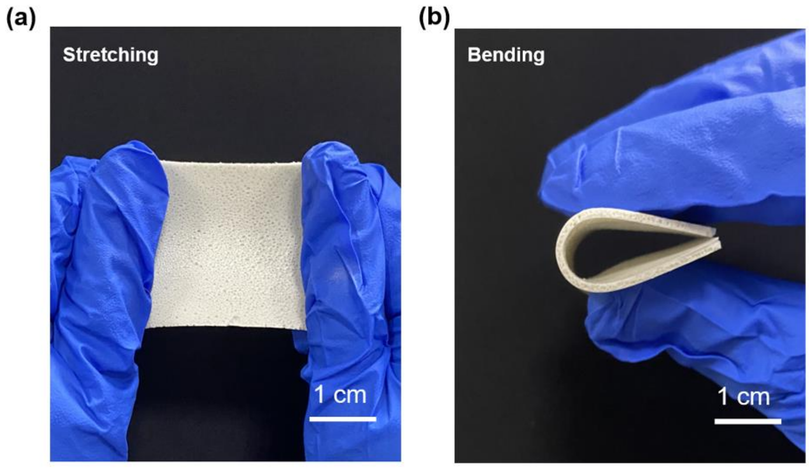

Figure S1. The photographs of the as-fabricated porous luminescent layer under a) stretching and b) bending.

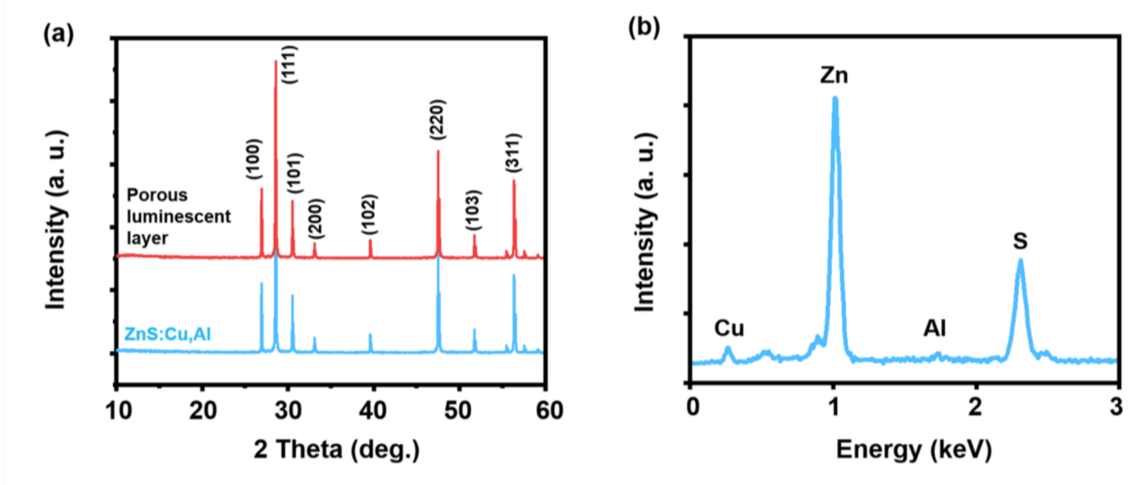

Figure S2. a) The XRD pattern of ZnS:Cu,Al phosphors and the as-fabreicated porous luminescent layer. b) EDS spectrum of ZnS:Cu,Al phosphors . Three main peaks at 2θ values of 28.5°, 47.5° and 56.3° corresponded to the (111), (220) and (311) lattice planes of the zinc-blended structure of ZnS (PDF#65-0309). The structure of ZnS:Cu,Al was not changed in the as-fabricated porous luminescent layer.

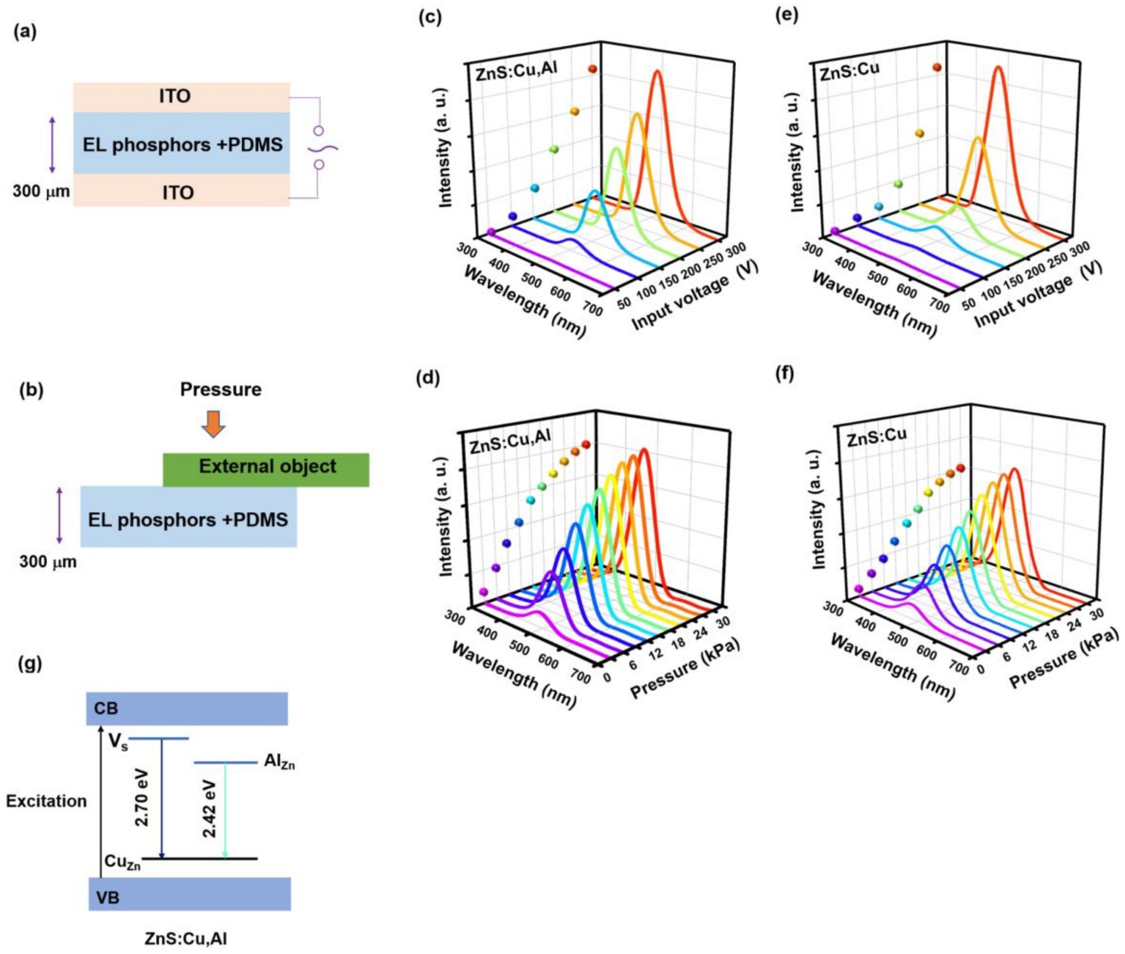

Figure S3. Comparison of EL and TIEL intensity between commercial ZnS:Cu,Al and ZnS: Cu phosphors (approximate in diameter). Schematic diagram of the characterization of a) EL and b) TIEL. The dependence of EL intensity of c) commercial ZnS:Cu,Al and e) ZnS:Cu under varied driven AC voltage at 50 Hz. Dependence of TIEL intensity of d) commercial ZnS:Cu, Al and f) ZnS:Cu under the varied contacted pressure of sliding stimuli. In this case, the thickness of dense EL phosphors/PDMS film was controlled at 300 μm for comparison. Finally, ZnS:Cu,Al was chosen in this case due to its higher luminous brightness than commercial ZnS: Cu because it is co-doped with Al as a co-activator.[17] g) Band diagram of the EL of ZnS:Cu, Al phosphor. The intense light emission of SPTS at 509 nm originates mostly from the D-A pairs recombination between AlZn-CuZn (2.42 eV).[45]

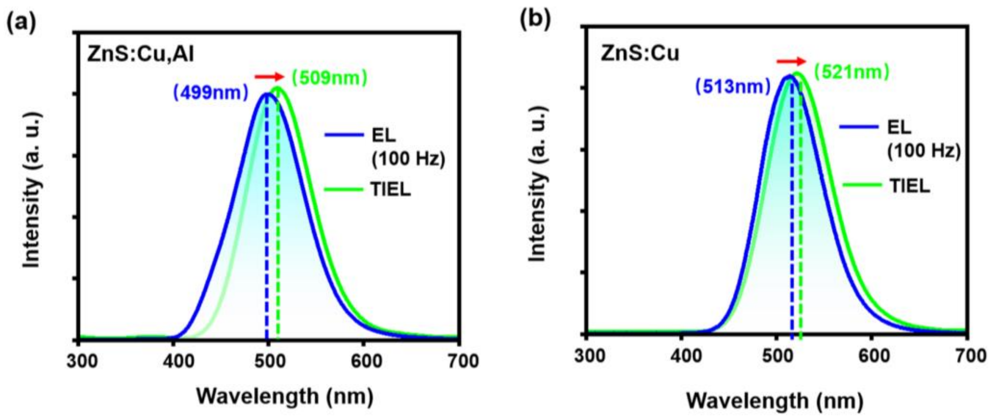

Figure S4. Spectrum shift between EL and TIEL due to the low frequency of TIEL.

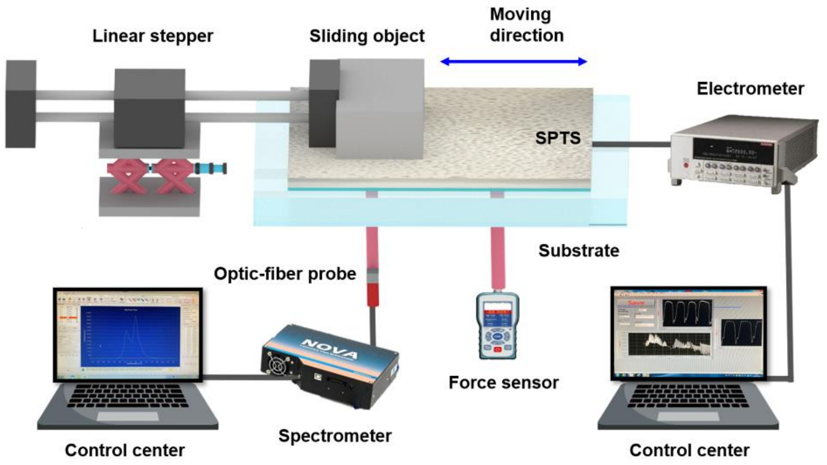

Figure S5. Schematic diagram of the test platform for the quantitative measurement of the optical and electrical output of the SPTS.

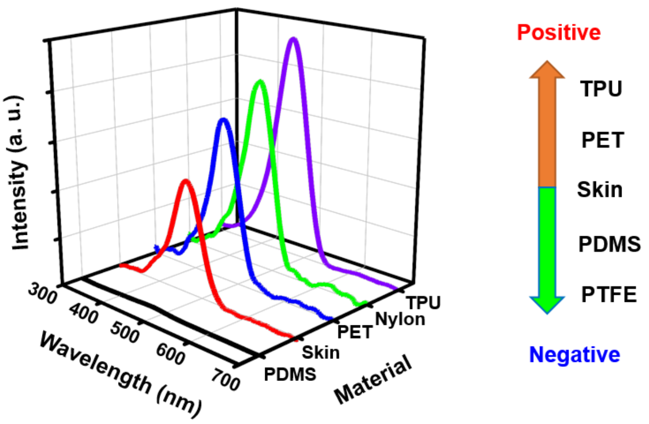

Figure S6. Dependence of wavelength spectra on the materials of the sliding object and the position of different materials in the "triboelectric series".

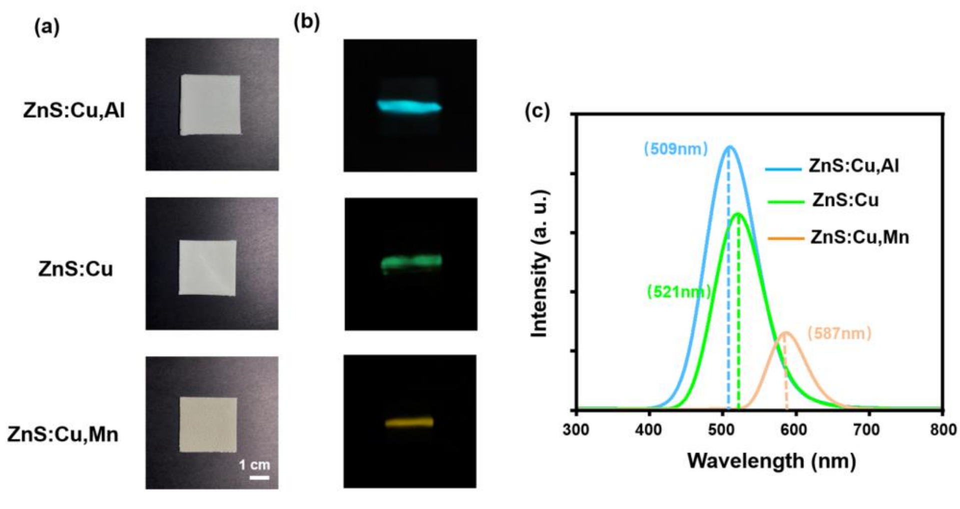

Figure S7. SPTS based on different EL powers (ZnS:Cu,Al; ZnS:Cu; and ZnS:Cu,Mn) displaying a variety of luminescent colors (green-blue, green, and orange). a) Actual photographs of the SPTS, b) luminescent photographs, and c) the measured spectrum of three SPTSs under sliding touch by finger.

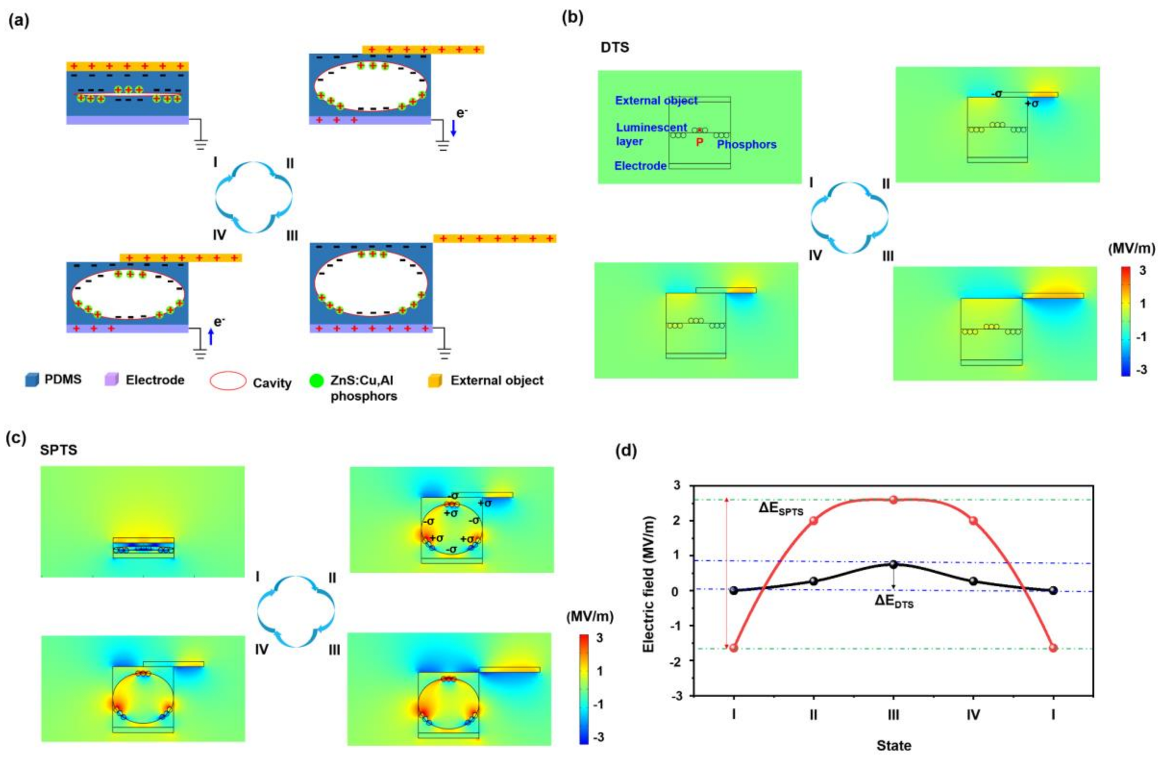

Figure S8. a) Simplified diagrams showing the process of TIEL and the electricity generation process of the SPTS under the sliding mode. The corresponding two-dimensional models of electric field distribution with the cross-section of b) DTS and c) SPTS under sliding mode. d) The extracted electric field of point "P" on the phosphor in the four typical working states of DTS and SPTS in the simulation. The electric field variation (ΔE) of phosphors in the DTS (at point P) is 0.75 MV/m in a period, as compared to 4.2 MV/m in the SPTS. The enhancement of the ΔE is comparable to that in the contact-separation mode (Figure 2d).

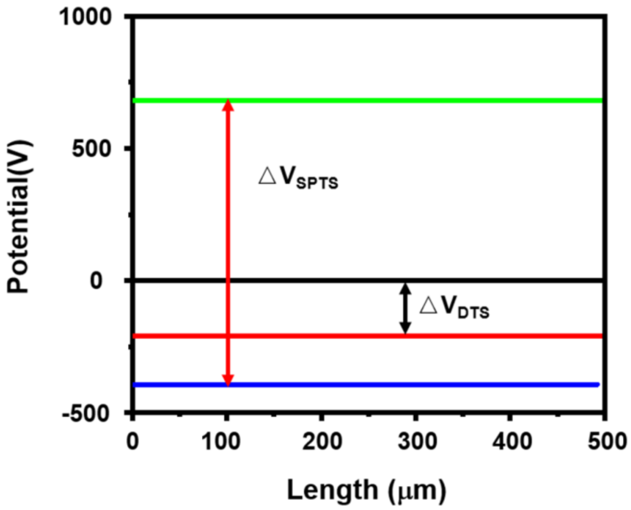

Figure S9. The potential value of the back electrode in the two states (I and III) of DTS and SPTS is indicated by the results of theoretical stimulation shown in Figure 2c), respectively.

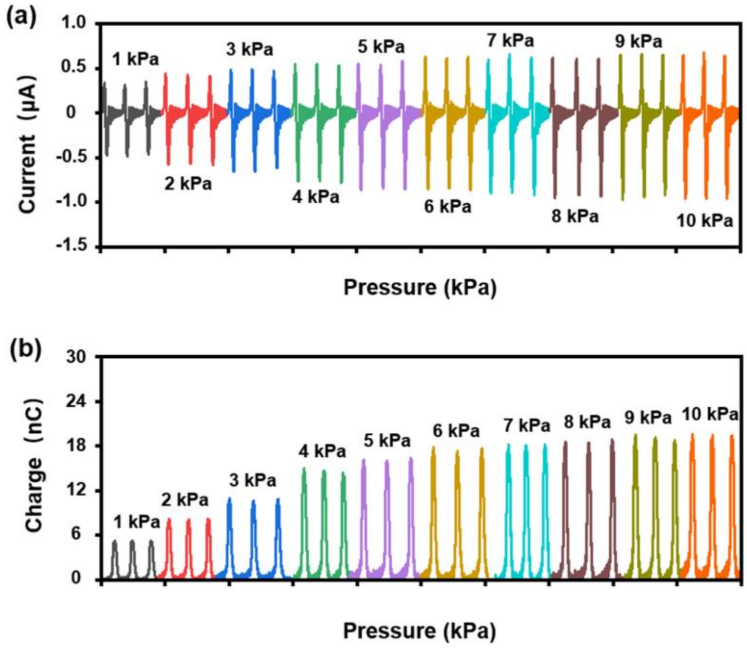

Figure S10. The current a) and transferred charge b) of SPTS under different pressures.

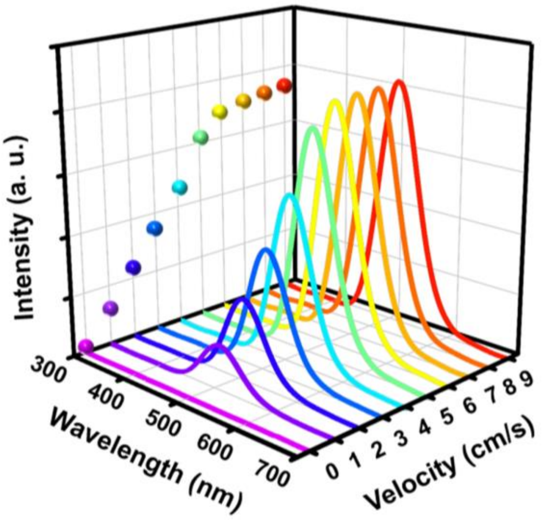

Figure S11. Dependence of wavelength spectra on the sliding velocity at a contact pressure of 5 kPa.

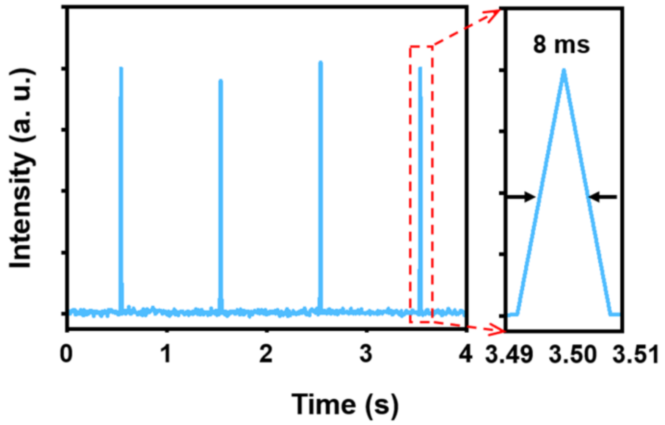

Figure S12. Response time of the SPTS. We slided on the surface of SPTS at a fast speed and set the integration time to 8 ms through the consecutive measurements of emission. Obviously, a TIEL peak will be obtained when the external object moves to the position of the device where the optical fiber locates on the other side of the device.

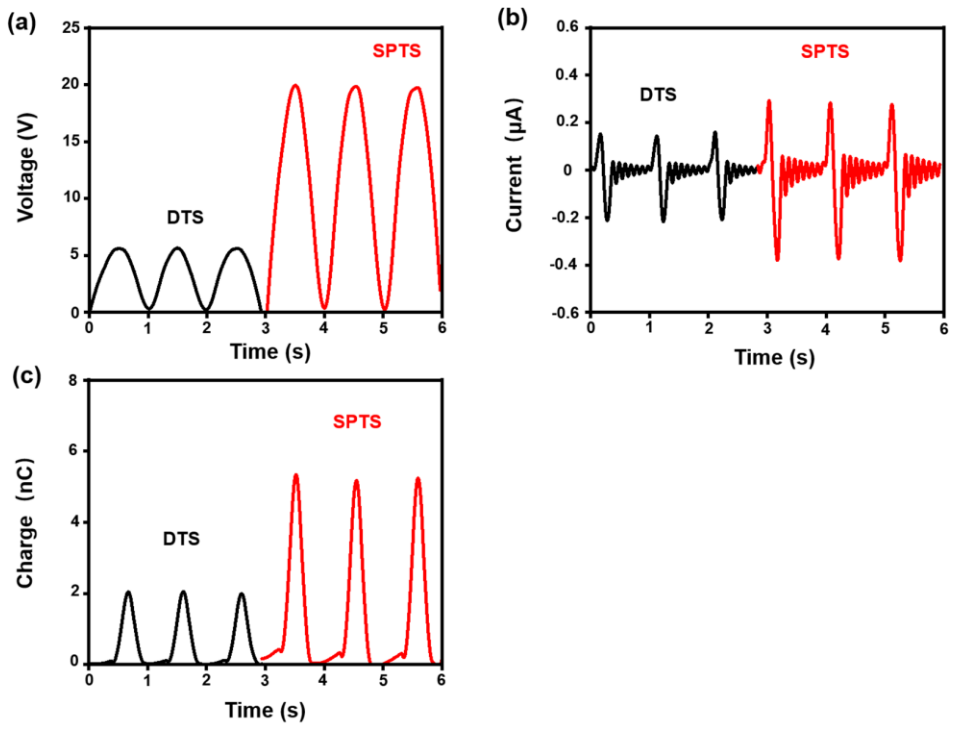

Figure S13. a) Voltage output, b) current and c) transferred charges of the DTS and SPTS under the sliding touch stimulus of the index finger.

Note 1: The calculation of porosity

The porosity (θ) can be calculated by dividing the volume of cavities in the SPTS by the total volume of SPTS, which can be calculated in the following equation[43,44]

$$\theta = \left(1 - \frac{M}{V\rho}\right) \times 100\% \quad (1)$$

where M and V represent the mass and volume of the the porous electroluminescent layer, respectively, ρ refers to the density of the cavity-free control sample. We controlled the porosity by varying the mass ratio between DI water to ZnS-PDMS composite during the fabrication process.

Note 2: The COMSOL simulation

The 2D models of SPTS and DTS were applied to demonstrate the distribution of electric potential and electric field. The PDMS matrix had a dimension of 500 μm×500 μm and an elliptical cavity with a height of 400 μm. The thickness of both the electrode and the contact object was set to 50 μm. The charge density of the PDMS and external object surface was set to 30 μC/m^2 and -30 μC/m^2, respectively, while that of three groups including ZnS:Cu,Al (40 μm) and PDMS on the inner cavity wall was set to 30 μC/m^2 and -30 μC/m^2, respectively. The dielectric permittivity used for simulation was ε = 1.0 for air and electrode, ε = 10.0 for external object, and ε = 20.0 for PDMS. The whole model was simulated in the presence of ambient air.
